# Ultra-Broadband High-Efficiency Solar Absorber Based on Double-Size Cross-Shaped Refractory Metals

**DOI:** 10.3390/nano10030552

**Published:** 2020-03-19

**Authors:** Hailiang Li, Jiebin Niu, Congfen Zhang, Gao Niu, Xin Ye, Changqing Xie

**Affiliations:** 1 Key Laboratory of Microelectronic Devices & Integrated Technology, Institute of Microelectronics, Chinese Academy of Sciences, Beijing 100029, China; niujiebin@ime.ac.cn; 2 College of Life Science and Engineering, Southwest University of Science and Technology, Mianyang 621010, China; Zhang2017@swust.edu.cn; 3 Research Center of Laser Fusion, China Academy of Engineering Physics, Mianyang 621900, China; toniugaooo@163.com

**Keywords:** solar energy absorber, refractory metal, propagating plasmon resonance, broadband absorption, FDTD

## Abstract

In this paper, a theoretical simulation based on a finite-difference time-domain method (FDTD) shows that the solar absorber can reach ultra-broadband and high-efficiency by refractory metals titanium (Ti) and titanium nitride (TiN). In the absorption spectrum of double-size cross-shaped absorber, the absorption bandwidth of more than 90% is 1182 nm (415.648–1597.39 nm). Through the analysis of the field distribution, we know the physical mechanism is the combined action of propagating plasmon resonance and local surface plasmon resonance. After that, the paper has a discussion about the influence of different structure parameters, polarization angle and angle of incident light on the absorptivity of the absorber. At last, the absorption spectrum of the absorber under the standard spectrum of solar radiance Air Mass 1.5 (AM1.5) is studied. The absorber we proposed can be used in solar energy absorber, thermal photovoltaics, hot-electron devices and so on.

## 1. Introduction

Electromagnetic metamaterials, a kind of composite material, is composed of the periodic arrangement of structural elements designed and manufactured manually. Since the electromagnetic metamaterials absorber can effectively absorb the light energy, and convert it into heat energy or other forms of energy, which has attracted great attention of scientists. Since Landy firstly proposed a narrow band perfect absorber, which was based on a metal-insulator-metal (MIM) in 2008 [1]. After that, more and more absorbers with high efficiency are proposed [2,3]. To date, there are many methods such as photovoltaic, solar cells, photothermal and hot spot generators can convert solar energy to other energy and application forms [4,5,6,7,8,9,10]. Hence, it is very important to utilize solar energy effectively. In subsequent studies, the metamaterials based on MIM are always designed to achieve the goal of single-band or multiband absorption [11,12,13,14,15]. However, in recent years, more and more researchers keep fixating on how to realize broadband absorption [16,17,18,19]. At present, some ways have been proposed and proved that they can be used to broaden the spectrum of the absorber by metal nanostructure. One of more classical methods is introducing two or more different nanoresonators into one unit of metamaterial at the same time [20,21,22,23,24,25]. The reason why it can broaden the absorption spectrum of the whole structure is that these different resonators are able to provide absorption peak at a different frequency. Another popular one is to stack metal-insular films to achieve broadband [26,27,28,29].

Compared with noble metals, refractory metals are more suitable for designing broadband absorption absorbers [30,31,32,33]. The reasons are listed in the following: Firstly, the reserves of refractory metals are larger than noble metals, and the price is more favorable than noble metals. Secondly, both noble and refractory metals have complex permittivity. In addition, their real parts are negative, which indicates they have the ability to maintain surface plasmon resonance (SPR). As we all know, the imaginary part of permittivity of metal determines the loss of light. That is to say, the larger the imaginary part of permittivity is, the greater the loss of light is. However, noble metals just can arouse very narrow absorption peaks owing to its small imaginary part of permittivity [34]. On the contrary, refractory metals can cause higher light absorption within broadband because of its large imaginary part of permittivity [35,36,37]. Thirdly, the melting point of refractory metal is high, which makes it withstand the high temperature produced by the absorber when it works. Since under the irradiation of strong incident light, the temperature of the absorber will be so high that the metal with lower melting point is easy to melt or even volatilize.

In our work, we choose refractory metal titanium (Ti) and titanium nitride (TiN) as metal materials of the absorber to make its plasma response be used better. In addition, we use SiO_2_ as the insulator of absorber. In addition, the method we choose is to introduce two or more different nanoresonators into one unit of metamaterial at the same time. The absorber we proposed can realize that the absorption is more than 90% in a spectrum of approximately 1182 nm wide. Besides, its supreme absorption is about 96%. In order to understand the physical mechanism, we simulate the electric field and magnetic field distributions. Then we study the effects of structure parameters, incident light angle and polarization angle on the absorber. Finally, we discuss the absorption effect of the absorber under the standard spectrum of solar radiance Air Mass 1.5 (AM1.5).

## 2. Structure Design and Numerical Model

**Figure 1:**
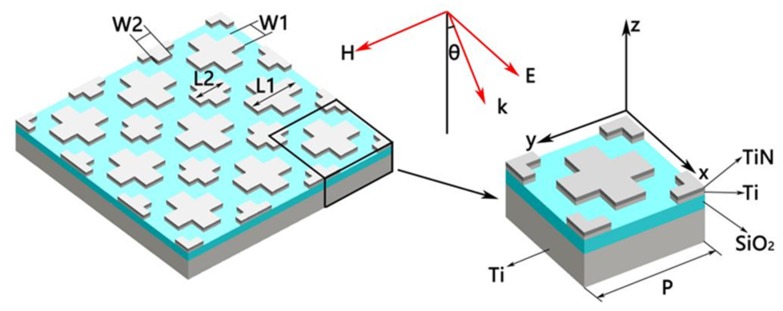
Three-dimensional stereogram of the double-size cross absorber.

Figure 1 shows the structure of the absorber we designed. The bottom metal is Ti. The insulator material in the middle is SiO_2_ (refractive index is 1.45) [38,39]. The top was composed of two cross-shaped nanostructures with different sizes. The cross-shaped material consisted of a layer of TiN and a layer of TiN. Then we defined the long and short axis of the big and small cross as *L*_1_, *L*_2_, *W*_1_ and *W*_2_, separately. Besides, the thickness of each layer of material from top to bottom was set to *t*_1_ (TiN), *t*_2_ (Ti), *t*_3_ (SiO_2_) and *t*_4_ (Ti). Additionally, the units of the absorber were arranged periodically with a period *P*. Later we named this absorber as a double-size cross-shaped absorber. The research method in this paper was the finite difference time domain (FDTD) [40,41]. Then we used the software FDTD Solutions (Lumerical Inc., Vancouver, BC, Canada) to model and simulate. The boundary conditions in the x and y directions of the structure were set to periodic boundary conditions. Additionally, the perfect matching layer is in the z direction. In addition, we set the mesh accuracy to 40 nm. This value can ensure that the calculated results were convergent and reliable. The incident light was set as a plane wave along the z direction. Moreover, the frequency-domain field and power monitors were used to collect the reflected and transmitted waves. Then we could get the reflection *R* from the monitor directly above the absorber and the transmission *T* from another monitor directly below the absorber. Besides, the absorption *A* would be obtained by the formula *A* = 1 − *R* − *T* [42,43,44,45,46]. What is more, we calculated the electric and magnetic field distributions by the frequency-domain field profile monitor. In this paper, we only considered transverse magnetic wave (TM wave). The permittivity of Ti and TiN were obtained from Palik’s experimental data [47]. In the experiment, the Si substrate was used to support the whole absorber. First of all, the Si substrate could be cleaned by ultrasonic with acetone and deionized water. After that, the Ti film of 190 nm, SiO_2_ film of 70 nm, Ti film of 20 nm and TiN film of 20 nm were respectively plated on the Si surface through magnetron sputtering. At last, the cross shape could be gained by standard photolithography [48].

## 3. Simulations Results and Discussions

**Figure 2:**
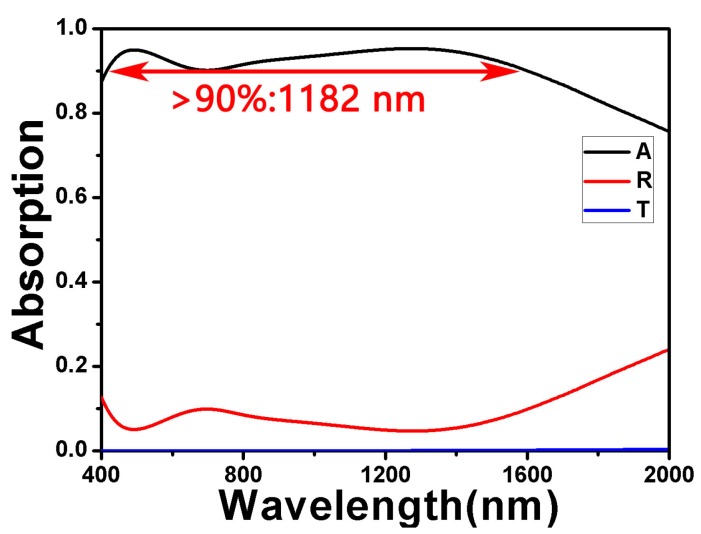
The absorption, reflectivity and transmittance spectra of the double-size cross-shaped absorber.

Firstly, by exploring several parameters of the double-size cross-shaped absorber, we obtained a set of parameters. This could make the absorber achieve that its absorption was more than 90% in nearly an 1182 nm wide spectrum. This set of parameters was *L*_1_ = 270 nm, *W*_1_ = 100 nm, *L*_2_ = 180 nm, *W*_2_ = 80 nm, *t*_1_ = 20 nm, *t*_2_ = 20 nm, *t*_3_ = 70 nm, *t*_4_ = 190 nm and *P* = 400 nm. Among them, the bottom Ti was thick enough to prevent light from passing through the absorber. As shown in Figure 2, we could see the absorption, reflectivity and transmittance spectra of the absorber under these structural parameters. Through formula *A* = 1 − *R* − *T*, we would obtain absorption *A*. The transmission of the absorber is almost zero because the transmission of light was hindered by the substrate Ti. Lower reflection and almost zero transmission lead to higher absorptivity. From 415.648 to 1597.39 nm in the spectrum, the absorptivity of the absorber was more than 90%. That is, the absorption bandwidth of more than 90% was 1182 nm. When the wavelength was 1276.83 nm, the absorption rate reached the highest, which was about 96%. In Table 1, we cited some examples of broadband high absorption using refractory metals in the past [49,50,51,52,53]. By comparison, we could conclude that the absorption effect of our absorber was better. 

**Figure 3:**
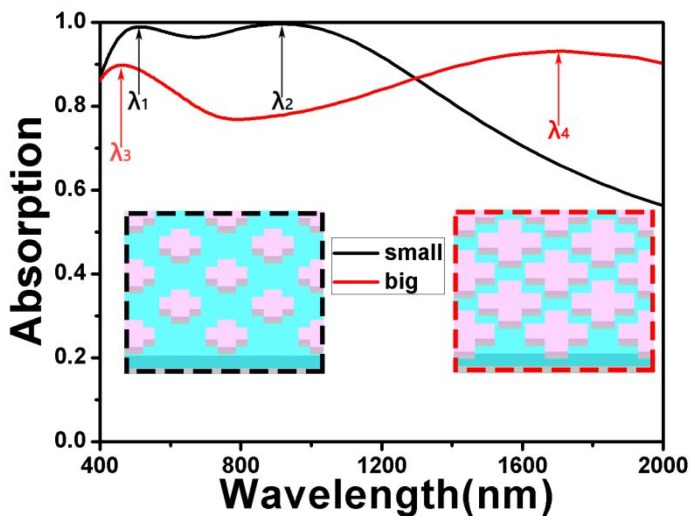
The absorption of two single-size cross-shaped absorbers and the inserts are three-dimensional image of two absorbers.

Then we simulated the absorption of two single-size cross-shaped absorbers, which is shown in Figure 3. The black line displays the absorption of the small cross with a long axis of 180 nm and a short axis of 80 nm. Additionally, the red line displays the absorption of the big cross with a long axis of 270 nm and a short axis of 100 nm. The inserts are three-dimensional image of two absorbers, respectively. From Figure 3, we can see that the absorption of big cross was more than 90% in the range of 500 nm. In addition, for the small cross, the absorption of more than 90% was concentrated in the short-wave band with a wave width of 850 nm. Here, it was easy to conclude that a single-size cross-shaped absorber could not achieve good results. However, we could broaden the absorption band by introducing two cross-shaped resonators with different sizes in the same cell. Next, we will discuss the physical mechanism behind it.

**Figure 4:**
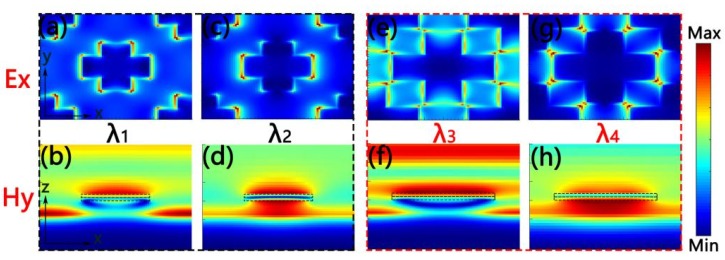
(**a**–**d**) are the distribution of electric fields (*Ex*) and magnetic fields (*Hy*) of the small cross-shaped absorber at *λ*_1_ and *λ*_2_. (**e**–**h**) are the distribution of electric fields (*Ex*) and magnetic fields (*Hy*) of the big cross-shaped absorber at *λ*_3_ and *λ*_4_.

In order to comprehend the physical mechanisms of the above two single-size cross-shaped absorbers, we calculated the electric and magnetic field distributions of them in λ_1_, λ_2_, λ_3_ and λ_4_, separately. Here, λ_1_ = 512.864 nm, λ_2_ = 911.585 nm, λ_3_ = 463.326 nm and λ_4_ = 1708.57 nm. Figure 4a is the electric field distribution of the small cross-shaped absorber. It can be seen that the electric field was localized at both ends of the horizontal axis and on both sides of the vertical axis. From Figure 4b, it is clear that the magnetic field was mainly distributed in the SiO_2_ buffer layer. These electric and magnetic field distributions indicate the existence of propagated plasmon resonance of the cross-shaped absorber. Propagated plasma resonance is generated by a lattice resonance, which is excited by periodic arrays [54]. At λ_2_, the electric field was strongly confined to both ends of the horizontal axis of the small cross-shaped absorber, which can be seen from Figure 4c. What is more, Figure 4d shows that the magnetic field was mainly confined between the cross-shaped Ti resonator and the substrate Ti. It also proves the existence of the local surface plasmon resonance excited by the cross resonator. Thus, the existence of the local surface plasmon resonance and plasmonic lattice resonance are the reasons for the broadband absorption of the small cross-shaped absorber [54]. The electric and magnetic field distribution of the big cross-shaped absorber at λ_3_ and λ_4_ are displayed in Figure 4e–h. Similarly, the cohesion of the local surface plasmon resonance and plasmonic lattice resonance caused two absorption peaks of the big cross-shaped absorber. The plasmon resonance stimulated by a cross resonator is closely related to the size of the resonator [55,56]. Therefore, the absorption spectra of these two cross-shaped absorbers with different sizes were different.

**Figure 5:**
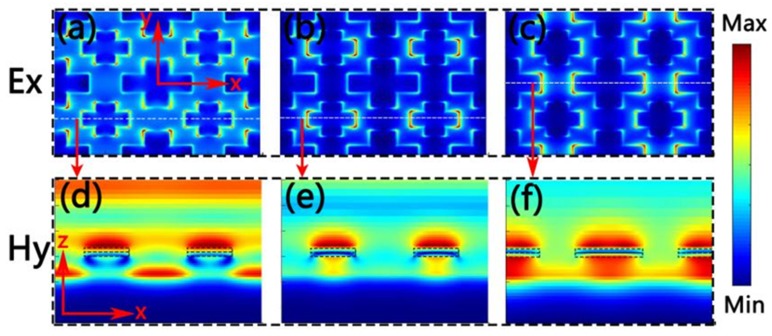
The electric field (*Ex*) and magnetic field (*Hy*) distributions of the double-size cross-shaped absorber at 492.11 nm (**a**,**d**), 697.78 nm (**b**,**e**) and 1276.83 nm (**c**,**f**), respectively.

Then we show the electric and magnetic field distributions of the double-size cross-shaped absorber at 492.11 nm, 697.78 nm and 1276.83 nm in Figure 5. This set of parameters was *L*_1_ = 270 nm, *W*_1_ = 100 nm, *L*_2_ = 180 nm, *W*_2_ = 80 nm, *t*_1_ = 20 nm, *t*_2_ = 20 nm, *t*_3_ =7 0 nm, *t*_4_ = 190 nm and *P* = 400 nm. When the resonant wavelength is 491.372 nm, Figure 5a,d are the distributions of electric and magnetic field, separately. We can see clearly that the electric field was mainly distributed at the top corner of the cross. In addition, the magnetic field was mainly confined to the SiO_2_. In addition, part of the magnetic field was confined to the TiN because the material itself absorbs light [57]. These field distributions in Figure 5 indicate the existence of propagated plasmon resonance and local surface plasmon resonance. Hence, we could say, local surface plasmon resonance and propagating plasmon resonance stimulated by cross-shaped resonators were the main reasons for the wide absorption band of double-size cross-shaped optical absorbers.

**Figure 6:**
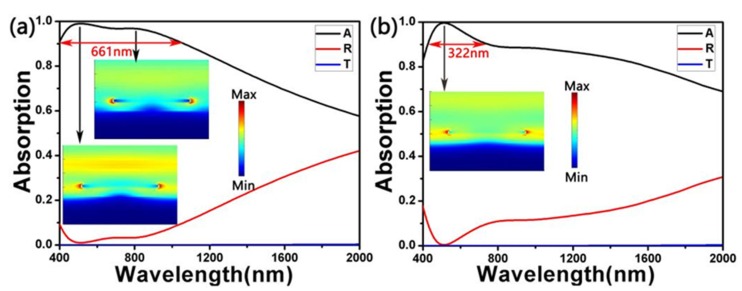
The spectra of a single layer of metal Ti (**a**) or TiN (**b**) on SiO_2_. Additionally, the inserts are their electric field distributions (*Ex*) at absorption peaks.

Moreover, we calculated the absorption of a single layer of metal (one layer of Ti or one layer of TiN) on SiO_2_, which are shown in Figure 6. Additionally, the insertion diagrams are the electric field distributions at the absorption peaks of the absorbers. This set of parameters was *L*_1_ = 270 nm, *W*_1_ = 100 nm, *L*_2_ = 180 nm, *W*_2_ = 80 nm, *t*_1_ = 20 nm, *t*_2_ = 20 nm, *t*_3_ = 70 nm, *t*_4_ = 190 nm and *P* = 400 nm. In Figure 6a, it is the absorption spectrum of the absorber with a single layer Ti on SiO_2_. Its absorption bandwidth of more than 90% was 661nm. There were two peaks in a short wavelength. At the first absorption peak (*λ* = 510.384 nm), the electric field was distributed at the top of the cross and at both ends of the cross, suggesting that local surface plasmons were responsible for the high absorption rate. At the second peak (*λ* = 771.613 nm), we could also see that the electric field was confined to the both ends of the cross. Figure 6b is the absorption spectrum of the absorber with a single layer TiN on SiO_2_. Its absorption bandwidth of more than 90% was 322 nm. In addition, the absorption peak appeared at 510.384 nm. Through Figure 6, we can know that when there was only one layer of metal Ti or TiN on the SiO_2_, the broadband and high absorption effect of the absorber was not as good as shown in Figure 2.

**Figure 7:**
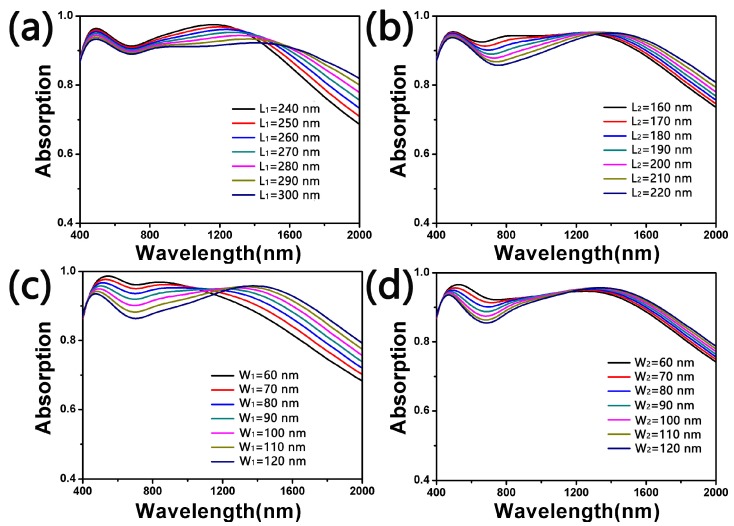
The absorption of the double-size cross-shaped absorber with different *L*_1_ (**a**), *L*_2_ (**b**), *W*_1_ (**c**) and *W*_2_ (**d**).

What is more, we discussed the absorption of the double-size cross-shaped absorber with different structural parameters in Figure 7. Here we only changed *L*_1_, *L*_2_, *W*_1_ and *W*_2_ while with *t*_1_ = 20 nm, *t*_2_ = 20 nm, *t*_3_ = 70 nm, *t*_4_ = 190 nm and *P* = 400 nm. When the length of the long axis *L*_1_ of the big cross resonator varies from 240 to 300 nm at intervals of 10 nm, its change of absorption is shown in Figure 7a. In addition, other parameters were set to *W*_1_ = 100 nm, *L*_2_ = 180 nm and *W*_2_ = 80 nm. As *L*_1_ = 240 nm, the two absorption peaks reached the maximum. With the increase of *L*_1_, the peak at a short wavelength dropped from 96.5% to 93.3%. Additionally, the peak at long wavelength fell by 5.5% (97.6–92.1%). In Figure 7b, we tuned *L*_2_ from 160 to 220 nm with *L*_1_ = 270 nm, *W*_1_ = 100 nm and *W*_2_ = 80 nm. The peak at short wavelength changed from 95.4% to 93.7%. In addition, the peak at a long wavelength had little change. With *L*_1_ = 270 nm, *L*_2_ = 180 nm and *W*_2_ = 80 nm, Figure 7c shows the peak at a short wavelength reduced from 98.6% to 93.5% and the peak at a long wavelength fluctuated between 95.1% and 97.7% as *W*_1_ increased from 60 to 120 nm. Last in Figure 7d, we adjusted *W*_2_ from 60 to 120nm with *L*_1_ = 270 nm, *L*_2_ = 180 nm and *W*_1_ = 100 nm. It can be seen that the peak at a short wavelength descended from 96.7% to 93.6% while the peak at a long wavelength had ascended by 1% (94.6–95.6%). These phenomena indicate that the plasmon resonance between the cross-shaped resonators was weakened. Thus, we could adjust the absorption spectrum by changing these parameters.

**Figure 8:**
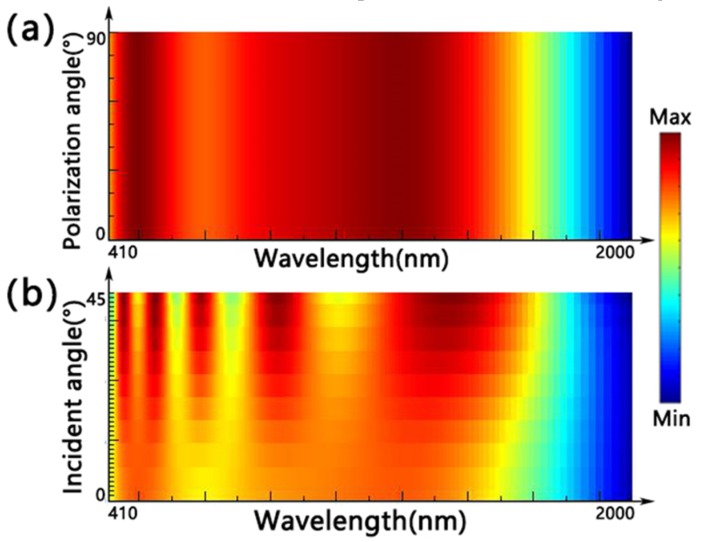
The effect of the different polarization angle (**a**) and incident angle (**b**) on the double-size cross-shaped absorber.

In addition, we studied the effects of the different polarization angle and incident angle on the double-size cross-shaped absorber in Figure 8. The set of parameters was *L*_1_ = 270 nm, *W*_1_ = 100 nm, *L*_2_ = 180 nm, *W*_2_ = 80 nm, *t*_1_ = 20 nm, *t*_2_ = 20 nm, *t*_3_ = 70 nm, *t*_4_ = 190 nm and *P* = 400 nm. We can see from Figure 8a that the absorber still kept the same absorption spectrum when the polarization angle rose from 0 (the direction of electric field along x axis) to 90° (the direction of electric field along x axis). The reason for this phenomenon is the symmetry of the unit of the absorber. Figure 8b shows how the incidence influences the absorption under TM polarization. It can be seen that the absorption band was broadening and the peaks were increasing with an additive incident angle, which was caused by the effective coupling between the oblique incidence and cross-shaped resonators. Consequently, the double-size cross-shaped absorber will maintain broadband and efficient absorption in the environment of variable polarization and incident angles.

**Figure 9:**
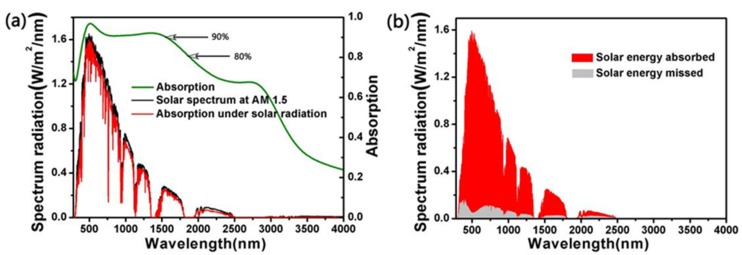
(**a**) The absorber’s absorption between 280 and 4000 nm (green line), the standard spectrum of solar radiance Air Mass 1.5 (AM1.5, black line) and the absorption spectrum of the absorber under the AM1.5 (red line). (**b**) The energy that absorber absorbs (red part) and misses (grey part) under the AM1.5.

At the last, we learnt the absorption effect of the absorber under the standard spectrum of solar radiance AM1.5. As is shown in Figure 9a, the green, black and red line are the absorber’s absorption between 280 and 4000 nm, the standard spectrum of solar radiance AM1.5 and the absorption spectrum of absorber under AM1.5, respectively [58,59,60,61]. It is clearly that the absorber had a high absorptivity in the band where the solar spectrum energy was concentrated, so its absorption spectrum under AM1.5 had a high fitting degree with AM1.5. For the sake of knowing more clearly the energy absorbed by the absorber in AM1.5, we show the energy that absorber absorbed (red part) and missed (grey part) under the AM1.5. Therefore, there is still a part of the energy that cannot be absorbed by the double-size cross-shaped absorber and lost.

## 4. Conclusions

In summary, we proposed a double-size cross-shaped absorber based on refractory metals Ti and TiN. Through adjusting several parameters, the absorber achieved that its absorption was more than 90% in a nearly 1182 nm wide spectrum. Then we obtained the main reason of its high broadband absorption through the analysis of the field distribution. It is the combined action of propagating plasmon resonance and local surface plasmon resonance. Moreover, we learnt a method of adjusting absorption spectra by changing structural parameters. What is more, the absorber was able to maintain broadband and efficient absorption in the environment of varying polarization and incident angles. In the end, we found that the absorber could absorb most of the solar energy, even if some of it was lost. Hence, the double-size cross-shaped absorber can be applied to solar energy absorber, thermal photovoltaics, hot-electron devices and so on.

## Figures and Tables

**Table 1:** Comparison between the different absorber designs proposed in previous studies [49,50,51,52,53].

Reference	Metallic Materials	Metal Patterning	Bandwidth with Absorptivity Greater than 90%
[49]	Cr	Closed-ring	660 nm
[50]	Cr	Without	1000 nm
[51]	TiN	Nanoellipsoid	400 nm
[52]	Ti	Square	712 nm
[53]	W	Meander-ring	459.22 nm
Present	Ti, TiN	Double-cross	1182 nm

## References

[1] Landy N.I., Sajuyigbe S., Mock J.J., Smith D.R., Padilla W.J. (2008). Perfect metamaterial absorber. Phys. Rev. Lett..

[2] Liang C.P., Yi Z., Chen X.F., Tang Y.J., Yi Y., Zhou Z.G., Wu X.G., Huang Z., Yi Y.G., Zhang G.F. (2020). Dual-band infrared perfect absorber based on a Ag-dielectric-Ag multilayer films with nanoring grooves arrays. Plasmonics.

[3] Wu P.H., Chen Z.Q., Jile H., Zhang C.F., Xu D.Y., Lv L. (2020). An infrared perfect absorber based on metal-dielectric-metal multi-layer films with nanocircle holes arrays. Results Phys..

[4] Khanna S., Newar S., Sharma V., Reddy K.S., Mallick T.K., Radulovic J., Khusainov R., Hutchinson D., Becerra V., Rufangura P. (2019). Electrical enhancement period of solar photovoltaic using phase change material. J. Clean Prod..

[5] Li J.K., Chen X.F., Yi Z., Yang H., Tang Y.J., Yi Y., Yao W.T., Wang J.Q., Yi Y.G. (2020). Broadband solar energy absorber based on monolayer molybdenum disulfide using tungsten elliptical arrays. Mater. Today Energy.

[6] Yi Z., Zeng Y., Wu H., Chen X.F., Fan Y.X., Yang H., Tang Y.J., Yi Y.G., Wang J.Q., Wu P.H. (2019). Synthesis, surface properties, crystal structure and dye-sensitized solar cell performance of TiO_2_ nanotube arrays anodized under different parameters. Results Phys..

[7] Granqvist C.G., Niklasson G.A. (2018). Solar energy materials for thermal applications: A primer. Sol. Energy Mater. Sol. Cells.

[8] Yu P.Q., Chen X.F., Yi Z., Tang Y.J., Yang H., Zhou Z.G., Duan T., Cheng S.B., Zhang J.G., Yi Y.G. (2019). A numerical research of wideband solar absorber based on refractory metal from visible to near infrared. Opt. Mater..

[9] Li J.K., Chen Z.Q., Yang H., Yi Z., Chen X.F., Yao W.T., Duan T., Wu P.H., Li G.F., Yi Y.G. (2020). Tunable broadband solar energy absorber based on monolayer transition metal dichalcogenides materials using Au nanocubes. Nanomaterials.

[10] Aydin K., Ferry V.E., Briggs R.M., Atwater H.A. (2011). Broadband polarization-independent resonant light absorption using ultrathin plasmonic super absorbers. Nat. Commun..

[11] Wu P.H., Chen Z.Q., Xu D.Y., Zhang C.F., Jian R.H. (2020). A narrow dual-band monolayer unpatterned graphene-based perfect absorber with critical coupling in the near infrared. Micromachines.

[12] Kou Z.Y., Miao C., Mei P., Zhang Y., Yan X.M., Jiang Y., Xiao W. (2020). Enhancing the cycling stability of all-solid-state lithium-ion batteries assembled with Li_1.3_Al_0.3_Ti_1.7_(PO_4_)_3_ solid electrolytes prepared from precursor solutions with appropriate pH values. Ceram. Int..

[13] Zhang W.B., Xiao Y. (2020). Mechanism of electrocatalytically active precious metal (Ni, Pd, Pt, and Ru) complexes in the graphene basal plane for ORR applications in novel fuel cells. Energy Fuels.

[14] Yan Y.X., Yang H., Yi Z., Xian T., Li R.S., Wang X.X. (2019). Construction of Ag_2_S@CaTiO_3_ heterojunction photocatalysts for enhanced photocatalytic degradation of dyes. Desalin. Water Treat..

[15] Wang S., Gao H., Sun G., Li Y., Wang Y., Liu H., Chen C., Yang L. (2020). Structure characterization, optical and photoluminescence properties of scheelite-type CaWO_4_ nanophosphors: Effects of calcination temperature and carbon skeleton. Opt. Mater..

[16] Li M.W., Liang C.P., Zhang Y.B., Yi Z., Chen X.F., Zhou Z.G., Yang H., Tang Y.J., Yi Y.G. (2019). Terahertz wideband perfect absorber based on open loop with cross nested structure. Results Phys..

[17] Li R., Miao C., Zhang M.Q., Xiao W. (2020). Novel hierarchical structural SnS_2_ composite supported by biochar carbonized from chewed sugarcane as enhanced anodes for lithium ion batteries. Ionics.

[18] Yan Y.X., Yang H., Yi Z., Wang X.X., Li R.S., Xian T. (2020). Evolution of Bi nanowires from BiOBr nanoplates through a NaBH_4_ reduction method with enhanced photodegradation performance. Environ. Eng. Sci..

[19] Wang Y.P., Jiang F.C., Chen J.F., Sun X.F., Xian T., Yang H. (2020). In situ construction of CNT/CuS hybrids and their application in photodegradation for removing organic dyes. Nanomaterials.

[20] Wang S., Chen C., Li Y., Zhang Q., Li Y., Gao H. (2019). Synergistic Effects of Optical and Photoluminescence Properties, Charge Transfer, and Photocatalytic Activity in MgAl_2_O_4_: Ce and Mn-Codoped MgAl_2_O_4_: Ce Phosphors. J. Electron. Mater..

[21] Wang J., He Z.B., Tan X.L., Wang T., Liu L., He X.S., Liu X.D., Zhang L., Du K. (2020). High-performance 2.6 V aqueous symmetric supercapacitor based on porous boron doped diamond via regrowth of diamond nanoparticles. Carbon.

[22] Yan Y.X., Yang H., Yi Z., Li R.S., Xian T. (2020). Design of ternary CaTiO_3_/g-C_3_N_4_/AgBr Z-scheme heterostructured photocatalysts and their application for dye photodegradation. Solid State Sci..

[23] Ni Y., Xiao W., Miao C., Xu M.B., Wang C.J. (2020). Effect of calcining oxygen pressure gradient on properties of LiNi_0.8_Co_0.15_Al_0.05_O_2_ cathode materials for lithium ion batteries. Electrochim. Acta.

[24] Wang Y.Y., Qin F., Yi Z., Chen X.F., Zhou Z.G., Yang H., Liao X., Tang Y.J., Yao W.T., Yi Y.G. (2019). Effect of slit width on surface plasmon resonance. Results Phys..

[25] Wang Y.Y., Chen Z.Q., Xu D.Y., Yi Z., Chen X.F., Chen J., Tang Y.J., Wu P.H., Li G.F., Yi Y.G. (2020). Triple-band perfect metamaterial absorber with good operating angle polarization tolerance based on split ring arrays. Results Phys..

[26] Cui Y., Fung K.H., Xu J., Ma H., Jin Y., He S., Fang N.X. (2012). Ultrabroadband light absorption by a sawtooth anisotropic metamaterial slab. Nano Lett..

[27] Cen C.L., Chen Z.Q., Xu D.Y., Jiang L.Y., Chen X.F., Yi Z., Wu P.H., Li G.F., Yi Y.G. (2020). High quality factor, high sensitivity metamaterial graphene-perfect absorber based on critical coupling theory and impedance matching. Nanomaterials.

[28] Yang M.M., Dai J.Y., He M.Y., Duan T., Yao W.T. (2020). Biomass-derived carbon from Ganoderma lucidum spore as a promising anode material for rapid potassium-ion storage. J. Colloid Interface Sci..

[29] Wu P.H., Zhang C.F., Tang Y.J., Liu B., Lv L. (2020). A perfect absorber based on similar fabry-perot four-band in the visible range. Nanomaterials.

[30] Liu G., Liu X., Chen J., Li Y., Shi L., Fu G., Liu Z. (2019). Near-unity, full-spectrum, nanoscale solar absorbers and near-perfect blackbody emitters. Sol. Energy Mater. Sol. Cells.

[31] Liang C.P., Zhang Y.B., Yi Z., Chen X.F., Zhou Z.G., Yang H., Yi Y., Tang Y.J., Yao W.T., Yi Y.G. (2019). A broadband and polarization-independent metamaterial perfect absorber with monolayer Cr and Ti elliptical disks array. Results Phys..

[32] Li H.L., Niu J.B., Wang G.Y. (2019). Dual-band, polarization-insensitive metamaterial perfect absorber based on monolayer graphene in the mid-infrared range. Results Phys..

[33] Wang J., Zhu M., Sun J., Yi K., Shao J. (2016). A broadband polarization-independent perfect absorber with tapered cylinder structures. Opt. Mater..

[34] Qin F., Chen Z.Q., Chen X.F., Yi Z., Yao W.T., Duan T., Wu P.H., Yang H., Li G.F., Yi Y.G. (2020). A Tunable Triple-Band Near-Infrared Metamaterial Absorber Based on Au Nano-Cuboids Array. Nanomaterials.

[35] Zeng Y., Chen X.F., Yi Z., Yi Y.G., Xu X.B. (2018). Fabrication of p-n heterostructure ZnO/Si moth-eye structures: Antireflection, enhanced charge separation and photocatalytic properties. Appl. Surf. Sci..

[36] He Z.H., Zhao J.L., Lu H. (2020). Tunable nonreciprocal reflection and its stability in a non-PT-symmetric plasmonic resonators coupled waveguide systems. Appl. Phys. Express.

[37] Naik G.V., Schroeder J.L., Ni X., Kildishev A.V., Sands T.D., Boltasseva A. (2012). Titanium nitride as a plasmonic material for visible and near-infrared wavelengths. Opt. Mater. Express.

[38] Wang S., Gao H., Chen C., Wei Y., Zhao X. (2019). Irradiation assisted polyacrylamide gel route for the synthesize of the Mg_1–x_Co_x_Al_2_O_4_ nano-photocatalysts and its optical and photocatalytic performances. J. Sol-Gel Sci. Technol..

[39] Wang Y.P., Yang H., Sun X.F., Zhang H.M., Xian T. (2020). Preparation and photocatalytic application of ternary n-BaTiO_3_/Ag/p-AgBr heterostructured photocatalysts for dye degradation. Mater. Res. Bull..

[40] Cen C.L., Zhang Y.B., Chen X.F., Yang H., Yi Z., Yao W.T., Tang Y.J., Yi Y.G., Wang J.Q., Wu P.H. (2020). A dual-band metamaterial absorber for graphene surface plasmon resonance at terahertz frequency. Physica E.

[41] Taflove A., Hagness S.C. (2005). Computational Electrodynamics: The Finite-Difference Time-Domain Method.

[42] Lv Y.R., Li Y.H., Han C., Chen J.F., He Z.X., Zhu J., Dai L., Meng W., Wang L. (2020). Application of porous biomass carbon materials in vanadium redox flow battery. J. Colloid Interface Sci..

[43] Zhang W.B., Xiao X., Wu Q.F., Fan Q., Chen S.J., Yang W.X., Zhang F.C. (2020). Facile synthesis of novel Mn-doped Bi_4_O_5_Br_2_ for enhanced photocatalytic NO removal activity. J. Alloys Compd..

[44] Guan S.T., Yang H., Sun X.F., Xian T. (2020). Preparation and promising application of novel LaFeO_3_/BiOBr heterojunction photocatalysts for photocatalytic and photo-Fenton removal of dyes. Opt. Mater..

[45] Wang S., Gao H., Chen C., Li Q., Li C., Wei Y., Fang L. (2019). Effect of phase transition on optical and photoluminescence properties of nano-MgWO_4_ phosphor prepared by a gamma-ray irradiation assisted polyacrylamide gel method. J. Mater. Sci. Mater. Electron..

[46] Yan Y.X., Yang H., Yi Z., Xian T. (2019). NaBH_4_-reduction induced evolution of Bi nanoparticles from BiOCl nanoplates and construction of promising Bi@BiOCl hybrid photocatalysts. Catalysts.

[47] Palik E.D. (1998). Handbook of Optical Constants of Solids.

[48] Wan C.L., Ho Y.L., Nunez-Sanchez S., Chen L.F., Lopez-Garcia M., Pugh J., Zhu B.F., Selvaraj P., Mallick T., Senthilarasu S. (2016). A selective metasurface absorber with an amorphous carbon interlayer for solar thermal applications. Nano Energy.

[49] Hu D., Wang H.Y., Zhu Q.F., Zhang X.W., Tang Z.J. (2016). Investigation of a broadband and polarization-insensitive optical absorber based on closed-ring resonator. J. Nonlinear Opt. Phys. Mater..

[50] Deng H., Li Z., Stan L., Rosenmann D., Czaplewski D., Gao J., Yang X. (2015). Broadband perfect absorber based on one ultrathin layer of refractory metal. Opt. Lett..

[51] Chen F., Li Q., Li M., Long H., Yao Y., Sun P., Zhang J. (2016). Simulation of perfect absorber at visible frequencies using TiN-based refractory plasmonic metamaterials. Opt. Quantum Electron..

[52] Lei L., Li S., Huang H., Tao K., Xu P. (2018). Ultra-broadband absorber from visible to near-infrared using plasmonic metamaterial. Opt. Express.

[53] Cao C., Cheng Y. (2019). A broadband plasmonic light absorber based on a tungsten meander-ring-resonator in visible region. Appl. Phys. A.

[54] Huan H., Jile H., Tang Y.J., Li X., Yi Z., Gao X., Chen X.F., Chen J., Wu P.H. (2020). Fabrication of ZnO@Ag@Ag_3_PO_4_ ternary heterojunction: Superhydrophilic properties, antireflection and photocatalytic properties. Micromachines.

[55] Xian T., Di L.J., Sun X.F., Li H.Q., Zhou Y.J., Yang H. (2019). Photo-Fenton degradation of AO7 and photocatalytic reduction of Cr(VI) over CQD-decorated BiFeO_3_ nanoparticles under visible and NIR light irradiation. Nanoscale Res. Lett..

[56] Li H.L., Wang G.Y., Niu J.B., Wang E.L., Niu G., Xie C.Q. (2019). Preparation of TiO_2_ nanotube arrays with efficient photocatalytic performance and super-hydrophilic properties utilizing anodized voltage method. Results Phys..

[57] Guler U., Boltasseva A., Shalaev V.M. (2014). Refractory plasmonics. Science.

[58] Wu H., Jile H., Chen Z.Q., Xu D.Y., Yi Z., Chen X.F., Chen J., Yao W.T., Wu P.H., Yi Y.G. (2020). Fabrication of ZnO@MoS_2_ nanocomposite heterojunction arrays and their photoelectric properties. Micromachines.

[59] Yi Z., Li X., Wu H., Chen X.F., Yang H., Tang Y.J., Yi Y., Wang J., Wu P.H. (2019). Fabrication of ZnO@Ag_3_PO_4_ core-shell nanocomposite arrays as photoanodes and their photoelectric properties. Nanomaterials.

[60] Wang S., Gao H., Wang Y., Sun G., Zhao X., Liu H., Chen C., Yang L. (2020). Effect of the sintering process on the structure, colorimetric, optical and photoluminescence properties of SrWO_4_ phosphor powders. J. Electron. Mater..

[61] Gueymard C.A., Myers D., Emery K. (2002). Proposed reference irradiance spectra for solar energy systems testing. Sol. Energy.

